# Synaptic plasticity in affective comorbidities of neuropathic pain: a bibliometric analysis based on WoSCC and PubMed (2003–2025)

**DOI:** 10.3389/fneur.2026.1916513

**Published:** 2026-09-03

**Authors:** Hao Wang, Lanting Huang, Zhigang Lin, Shuijin Chen, Lechun Chen, Hongye Huang, Huanzhen Zhang, Chenyu Wang

**Affiliations:** 1 First Clinical Medical College, Fujian University of Traditional Chinese Medicine, Fuzhou, China; 2 Affiliated Rehabilitation Hospital of Fujian University of Traditional Chinese Medicine, Fuzhou, China; 3 Fujian Key Laboratory of Rehabilitation Technology, Fuzhou, China

**Keywords:** affective comorbidities, anterior cingulate cortex, bibliometric analysis, long-term depression, long-term potentiation, neuropathic pain, synaptic plasticity

## Abstract

**Objective:**

Neuropathic pain (NP) is frequently accompanied by anxiety and depression, creating a vicious cycle that impairs quality of life. Synaptic plasticity—particularly long-term potentiation (LTP) and long-term depression (LTD)—is increasingly recognized as a shared neural substrate linking NP to affective comorbidities. This bibliometric analysis maps the research landscape at this intersection.

**Methods:**

Data were retrieved from the Web of Science Core Collection (January 1, 2003, to December 31, 2025) and PubMed (2003 to 2025). After deduplication and eligibility screening, a total of 487 unique publications were identified. Citation metrics and visualization analyses were performed using VOSviewer, CiteSpace, and Bibliometrix.

**Results:**

Publication output has grown steadily since 2003, peaking in 2025 (43 studies). China leads in publication count (180), while the United States holds the highest citation impact (9,723 citations; 64.82 per study). Air Force Medical University and the Xi’an Jiaotong University are core institutions. *PAIN* (24 studies) and Journal of Neuroscience (22 studies) are the leading journals. Keyword and co-citation analyses indicate that research themes have undergone a phased transition from spinal cord mechanisms to limbic circuits, and then to the comorbidity mechanisms of pain and affective comorbidities, with a gradual shift toward clinical translation.

**Conclusion:**

This bibliometric analysis maps a field advancing from single-region synaptic mechanisms toward multi-region circuit integration, with growing contributions from immunology. Persistent gaps in sex-difference characterization and longitudinal study design mark priorities for future investigation. As a descriptive exercise bounded by two databases, these findings should be weighed accordingly.

## 1 Introduction

Neuropathic pain (NP) is caused by damage or disease of the somatosensory nervous system, manifesting as chronic pain with spontaneous pain, hyperalgesia, and paresthesia. A defining feature of NP is its frequent comorbidity with anxiety and depression, which creates a self-reinforcing cycle that substantially reduces patients’ quality of life (1, 2).

Synaptic plasticity, particularly long-term potentiation (LTP) and long-term depression (LTD), forms the cellular basis of learning and memory and is also involved in central sensitization processes in pain. Studies have shown that peripheral nerve injury can induce abnormal LTP or LTD in pain-related regions, such as the dorsal horn of the spinal cord, the anterior cingulate cortex (ACC), the amygdala, and the insular cortex (3–8). Changes in synaptic plasticity involve the transport and functional regulation of N-methyl-D-aspartate receptor (NMDAR) and α-amino-3-hydroxy-5-methyl-4-isoxazolepropionic Acid Receptor (AMPA) receptor subunits (8, 9), downstream signaling pathways (e.g., cAMP-PKA in AC1), and glial-neuronal interactions (10, 11).

**Figure 1:**
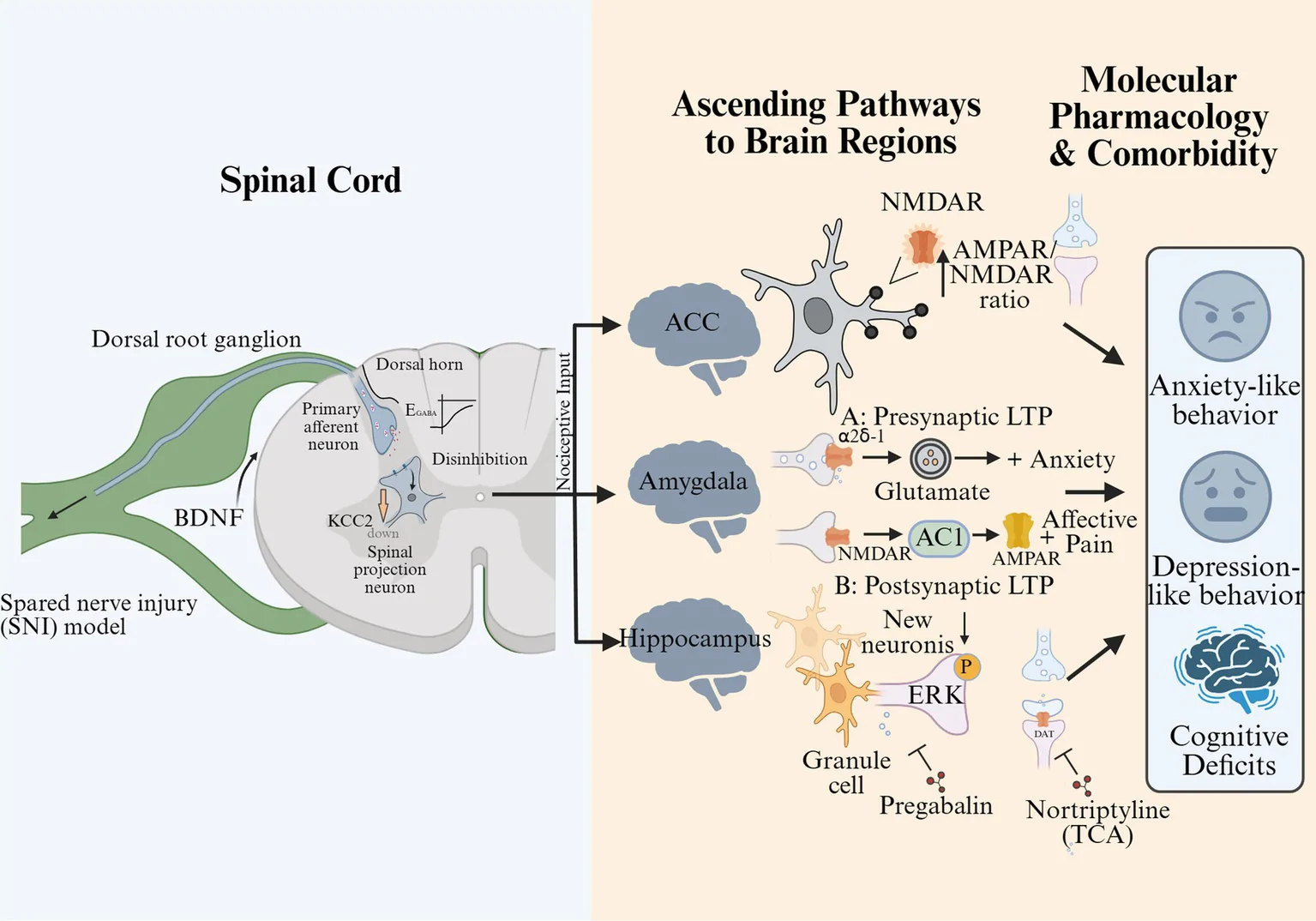
Synaptic plasticity mechanisms in neuropathic pain with emotional comorbidity. Created by BioRender. (DRG, Dorsal Root Ganglion; SNI, Spared nerve injury; mPFC, Medial Prefrontal Cortex; ACC, Anterior Cingulate Cortex; BDNF, Brain-Derived Neurotrophic Factor; NMDAR, N-methyl-D-aspartate receptor; AMPAR, α-amino-3-hydroxy-5-methyl-4-isoxazolepropionic acid receptor; ERK, Extracellular Signal-Regulated Kinase; AC1, Adenylyl Cyclase 1; α₂δ, Voltage-Gated Calcium Channel α₂δ Subunit; LTP, Long-Term Potentiation; KCC2, K^+^-Cl^−^ Cotransporter 2; E_GABA_, GABA Equilibrium Potential).

In recent years, the focus of research on NP has gradually shifted from the spinal cord to cognitive and emotion-related brain regions. Alterations in synaptic plasticity in these regions are now considered central neural mechanisms underlying the development and maintenance of NP and its associated emotional comorbidity (Figure 1) (3, 4).

For example, chronic pain impairs hippocampal neurogenesis and synaptic plasticity, leading to memory deficits and depression-like behaviors (12, 13). Similarly, reduced GABAergic inhibition and enhanced glutamatergic transmission within the amygdala are directly associated with pain-related anxiety-like behaviors (6).

Although numerous studies have revealed various links between NP, emotional comorbidity, and synaptic plasticity, insights into the patterns of scientific productivity, thematic evolution, collaborative networks, and knowledge dissemination in this field remain limited. Therefore, this study aims to utilize bibliometric methods to explore the current state of research on emotional comorbidity and synaptic plasticity in NP, evaluating scientific output, citation performance, collaboration patterns, and thematic evolutions.

## 2 Materials and methods

### 2.1 Eligibility criteria and screening

The inclusion criteria were as follows: (1) studies investigating synaptic plasticity mechanisms in the context of neuropathic pain with emotional comorbidity (anxiety and/or depression); (2) records with complete bibliographic information; (3) publicly published articles or reviews; (4) English-language publications.

The exclusion criteria were reports: (1) meeting abstracts, corrections, early access articles, editorial material, proceedings papers, book chapters, letters, news items, biographical items, and other non-research documents; (2) incomplete or unavailable records; (3) duplicate publications; (4) non-English records.

### 2.2 Literature sources and search strategy

After identifying relevant title keywords and supplementing them with Medical Subject Headings (MeSH) terms from PubMed, we conducted a comprehensive literature search of the Web of Science Core Collection (WoSCC) and PubMed using the search strategy outlined in Table 1. The search period was restricted to publications from January 1, 2003, to December 31, 2025. The search date was August 1, 2026. WoSCC was selected as the core bibliometric database because it provides a relatively comprehensive collection of citation indexes, cited references, journal information, and institutional affiliations. PubMed was used as a supplementary biomedical database to enhance coverage of clinical and medical literature.

**Table 1:** Search strategy.

Search strategies	
Databases	
PubMed	(“Neuralgia”[Mesh] OR “Sciatica”[Mesh] OR “Peripheral Nervous System Diseases”[Mesh] OR “Diabetic Neuropathies”[Mesh] OR Neuralgia[Title/Abstract] OR Neurodynia[Title/Abstract] OR “Neuropathic pain”[Title/Abstract] OR sciatica[Title/Abstract] OR “nerve crush”[Title/Abstract] OR “Nerve pain”[Title/Abstract] OR “nerve cut”[Title/Abstract] OR “nerve constriction”[Title/Abstract] OR “nerve inflammation”[Title/Abstract] OR “nerve injury”[Title/Abstract] OR “nerve ligation”[Title/Abstract] OR “peripheral neuropathy”[Title/Abstract] OR “chronic constriction injury”[Title/Abstract] OR “diabetic neuropathy”[Title/Abstract]) AND (“Depression”[Mesh] OR “Depressive Disorder”[Mesh] OR “Anxiety”[Mesh] OR “Anxiety Disorders”[Mesh] OR depression[Title/Abstract] OR “depressive symptom”[Title/Abstract] OR depressive[Title/Abstract] OR anxiety[Title/Abstract] OR “anxiety disorder”[Title/Abstract]) AND (“Synaptic Transmission”[Mesh] OR “Long-Term Potentiation”[Mesh] OR “Neuronal Plasticity”[Mesh] OR synap*[Title/Abstract] OR “synaptic plasticity”[Title/Abstract] OR “synaptic transmission”[Title/Abstract] OR “synaptic dysfunction”[Title/Abstract] OR “long-term potentiation”[Title/Abstract] OR “long-term depression”[Title/Abstract])
Web of Science Core Collection	(TS = (Neuralgia* OR Neurodynia* OR “Neuropathic pain” OR sciatica OR “nerve crush” OR “nerve pain*” OR “nerve cut” OR “nerve constriction” OR “nerve inflammation” OR “nerve injury” OR “nerve ligation” OR “peripheral neuropathy” OR “chronic constriction injury” OR “diabetic neuropathy”)) AND (TS = (depression OR depressive symptom OR depressive OR anxiety OR anxiety disorder)) AND (TS = (“synap*” OR “synaptic plasticity” OR “synaptic transmission” OR “synaptic dysfunction” OR “long-term potentiation” OR “long-term depression”))

### 2.3 Data processing

**Figure 2:**
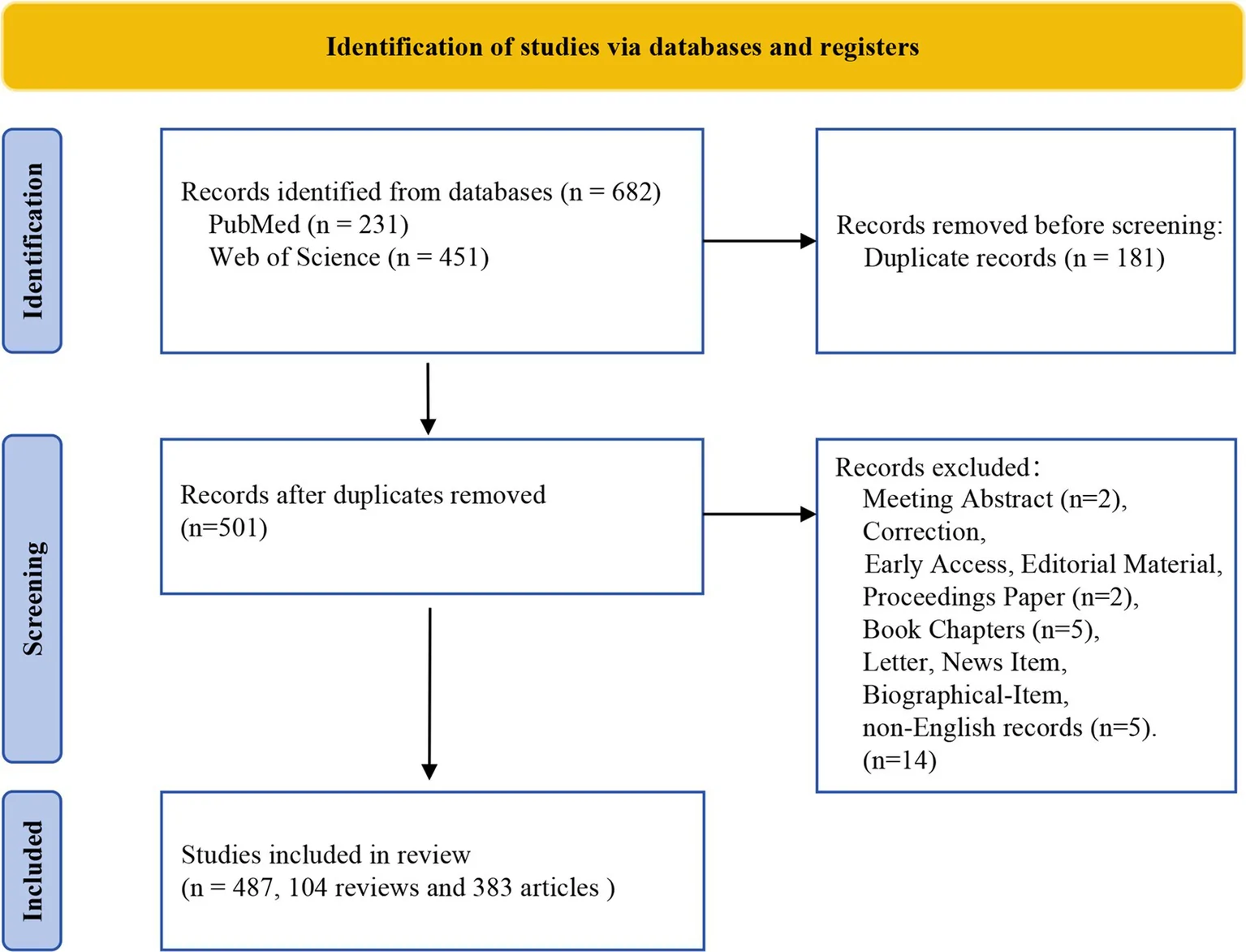
Flow diagram of the included studies.

WoSCC records were exported as “Full Record and Cited References” in plain text format, and PubMed records were exported in PubMed. WoSCC and PubMed generated 451 and 231 potential relevant records before deduplication. It is essential to verify that the exported txt file contains all specified fields. For example, the CR field must contain the reference list. Subsequently, data cleaning was performed to check the completeness and accuracy of all key fields, including title, author, year, keywords, and references. Records lacking core information, containing unrecognizable characters, or with incomplete reference data were excluded. Zotero 9.0.6 and the R bibliometrix package were used for data conversion, merging, duplicate removal and completeness checking. Duplicate content is removed primarily by matching DOIs, PMIDs, titles, and authors, with priority given to DOIs. At the same time, titles, abstracts, and keywords were manually reviewed to ensure that the records covered the three core areas of this study. Records that did not meet these requirements were excluded. After merging the databases and removing duplicate records, a total of 487 records were retained for bibliometric analysis, including 104 reviews and 383 articles; of these, 441 were from WoSCC and 46 were from PubMed. When the same publication appeared in both databases, the WoSCC version was retained because it contained more complete citation metadata. Since PubMed lacks complete citation information, its data were used only for calculating statistics on national, institutional, and author output. A detailed flowchart of the search and selection process is presented in Figure 2.

Prior to visualization, the data were standardized. The country labels appearing in CiteSpace-generated network maps are rendered automatically from the WoSCC (e.g., “United States,” “PEOPLES R CHINA”). In the manuscript text and manually prepared tables, country names have been standardized to their conventional English forms (e.g., “United States,” “China”). The author names were standardized by harmonizing initials, spelling variations, and punctuation. Institutional affiliations were manually verified to merge different representations of the same institution, including abbreviations and historical names. Keywords were standardized using a thesaurus and manual review. For example, “long-term potentiation (ltp)” was merged with “long term potentiation.”

### 2.4 Bibliometric and visualization analysis

Two researchers (WH and HLT) independently verified the raw WoSCC and PubMed data. In the event of any discrepancies, a third party conducted a re-evaluation, and a consensus among the three parties was reached immediately. CiteSpace (v6.4), VOSviewer (v1.6.20), R software 4.5.3 and the bibliometrix package 5.4.1 were used for analysis. VOSviewer (v1.6.20) was used to visualize co-occurrence networks for highly cited literature, authors, and keywords (14, 15). CiteSpace (v6.4) generated visualization maps by country, institution, author, and keyword, and performed burst detection on keywords and cited references. Aggregated statistics were computed via the online bibliometric platform^1^.

The H-index was used to evaluate the sustained influence of authors or journals. Centrality was used to identify bridging nodes in the network. Total link strength was used to represent the overall strength of collaboration, co-occurrence or co-citation links between a node and other nodes. Full counting was applied in network analyses. In CiteSpace, the time slice was set to 1 year, and node types were selected according to the analysis objective, including country, institution, author, reference or keyword. Thresholds were set as Top *N* = 50 or g-index with *k* = 25. Cluster labels were generated using the log-likelihood ratio algorithm, and network pruning was performed using Pathfinder or pruning sliced networks. Keyword cluster quality was evaluated using modularity *Q* and mean silhouette *S* values; *Q* > 0.3 indicated a significant cluster structure, *S* > 0.5 indicated acceptable clustering and *S* > 0.7 indicated high reliability. The VOSviewer clustering resolution is set to 1.0, and the minimum cluster size is set based on the specific nodes.

## 3 Results

### 3.1 Trends in publications and citations

**Figure 3:**
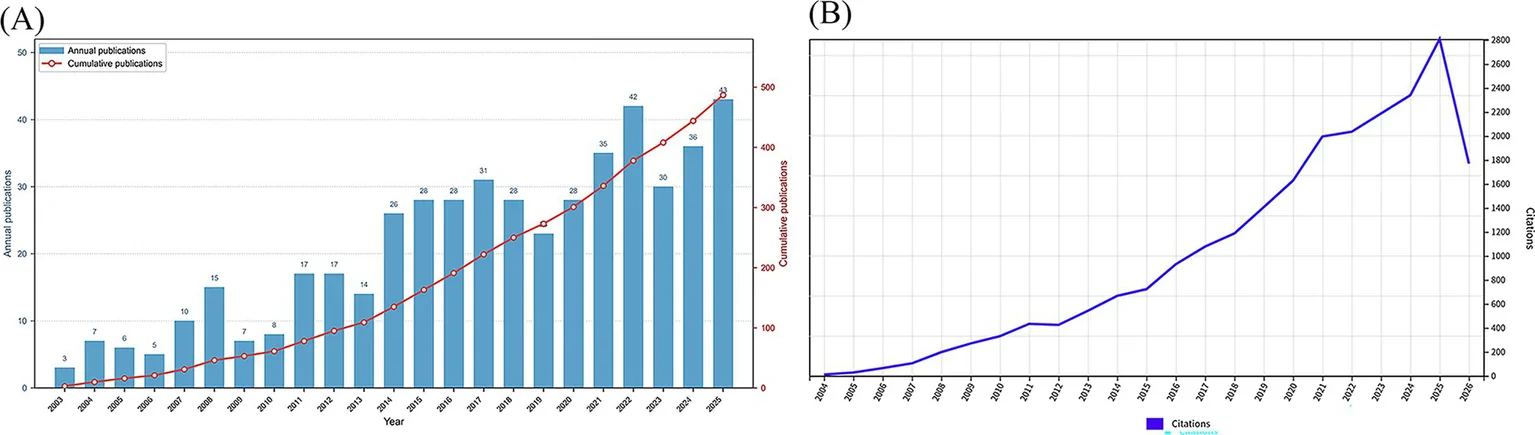
Publication trends in synaptic plasticity mechanisms of neuropathic pain with emotional comorbidity research. **(A)** Annual and cumulative number of publications. **(B)** Annual number of citations.

After screening, 487 studies were identified (Figure 3). The earliest relevant publication appeared in 2003. From 2003 to 2025, annual publication output showed a steady upward trend, peaking at 43 studies in 2025. After a brief dip to 7 studies in 2009, output recovered and accelerated from 2010 onward. Citations exhibited accelerating growth, rising sharply from approximately 900 in 2016 to approximately 2,800 in 2025. These trends collectively signal strong and sustained growth in both research activity and academic influence.

### 3.2 National and institutional analyses

**Figure 4:**
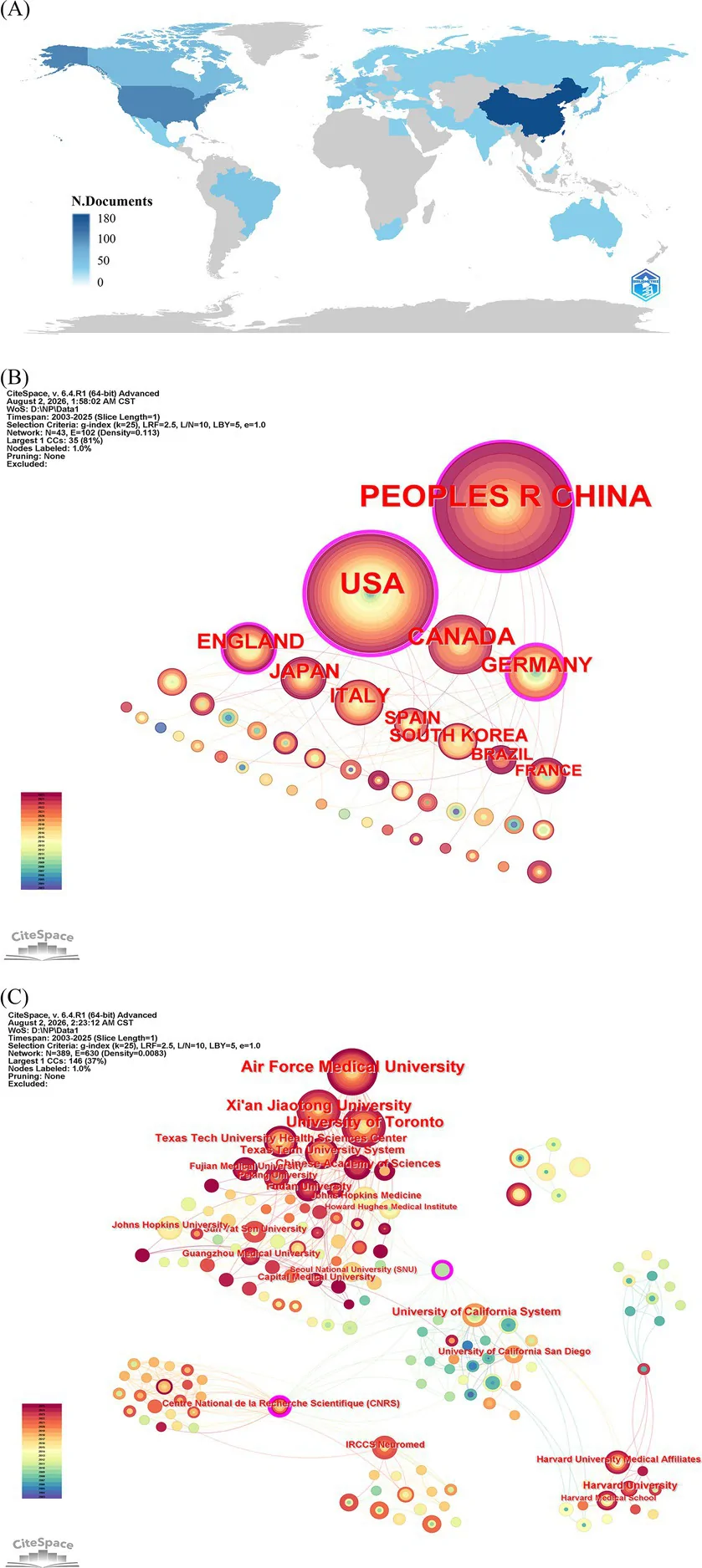
**(A)** Comparative map of the cumulative number of studies published in each country. Created with Bibliometrix. **(B)** Country/region collaboration network of research on synaptic plasticity mechanisms of neuropathic pain with emotional comorbidity. Created with CiteSpace. **(C)** Institution collaboration network of research on synaptic plasticity mechanisms of neuropathic pain with emotional comorbidity. Created with CiteSpace.

China and the United States together contributed 330 studies, with China ranking first in output (180 studies) and the United States ranking first in citation impact (9,723 citations; 64.82 per study) and network centrality (0.6) (Table 2, Figures 4A,B). Among 738 contributing institutions, Chinese institutions occupy four of the top ten positions by output. Air Force Medical University, Xi’an Jiaotong University, and the University of Toronto lead in publication volume (Table 3; Figure 4C). However, centrality analysis revealed a “production–hub” divergence: the University of Bordeaux (0.13) and Centre National de la Recherche Scientifique (CNRS) (0.11) serve as the largest network hubs, while United States institutions (University of California system, 0.10; David Geffen School of Medicine at UCLA, 0.06; Johns Hopkins University, 0.04) collectively anchor the United States national-level centrality advantage (0.6). China’s strength lies in large-scale knowledge output; the United States and France lead in establishing and coordinating international collaborative networks. This separation of production and hub roles is a defining structural feature of the current research landscape.

**Table 2:** The top 10 countries ranked by publications and centrality (Based on 487 publications).

Rank	Country	NP	TC (%)	AC	Country	Centrality
1	China	180	7,051 (19.06%)	39.17	United States	0.6
2	United States	150	9,723 (26.28%)	64.82	Germany	0.2
3	Canada	54	4,072 (11.01%)	75.41	China	0.19
4	Germany	31	1,461 (3.95%)	47.13	England	0.17
5	England	29	3,734 (10.09%)	128.76	Italy	0.1
6	Japan	28	999 (2.70%)	35.68	Norway	0.08
7	Italy	27	1,300 (3.51%)	48.15	Spain	0.06
8	Spain	18	593 (1.60%)	32.94	Canada	0.05
9	South Korea	17	1,617 (4.37%)	95.12	South Korea	0.05
10	Brazil	14	377 (1.02%)	26.93	France	0.05

**Table 3:** The top 10 institutions in synaptic plasticity mechanisms of neuropathic pain with emotional comorbidity research ranked by publications and centrality (Based on 487 publications).

Rank	Institution	NP	Country	Institutions	Centrality	Country
1	Air Force Medical University	37	China	Universite de Bordeaux	0.13	France
2	Xi’an Jiaotong University	31	China	Centre National de la Recherche Scientifique (CNRS)	0.11	France
3	University of Toronto	29	Canada	University of California System	0.10	United States
4	Texas Tech University Health Sciences Center Lubbock	11	United States	David Geffen School of Medicine at UCLA	0.06	United States
5	Texas Tech University System	11	United States	Air Force Medical University	0.05	China
6	Chinese Academy of Sciences	10	China	Xi’an Jiaotong University	0.04	China
7	University of California System	10	United States	Johns Hopkins University	0.04	United States
8	Fudan University	9	China	Howard Hughes Medical Institute	0.04	United States
9	Harvard University	8	United States	Laval University	0.04	Canada
10	Centre National de la Recherche Scientifique (CNRS)	7	France	Duke University	0.04	United States

### 3.3 High-contributing journals

The top 10 journals by publication volume account for 140 studies (28.75%) (Table 4). *PAIN* (2025 JCR Q1, IF = 5.5) leads with 24 studies, while *Journal of Neuroscience* ranks second (22 studies) but first in total citations (1,902). Notably, several journals with low publication counts carry disproportionately high citation impact: *Pharmacological Reviews* (910 citations, 1 study), *Brain* (846 citations, 1 study), *Nature Reviews Neuroscience* (676 citations, 1 study), and *Nature* (529 citations, 1 study) (4, 16–18), indicating that landmark studies in this field appear across a broad spectrum of high-impact venues.

**Table 4:** The top 10 related popular journals (Based on 487 publications).

Rank	Journal	NP	TC	AC	h_index	IF-2025	JCR
1	PAIN	24	769	32.04	16	5.5	Q1
2	Journal of Neuroscience	22	1902	86.45	20	4.4	Q2
3	Molecular Pain	18	513	28.50	11	2.9	Q3
4	Neuropharmacology	16	424	26.50	11	4.6	Q1
5	Molecular Brain	16	508	31.75	12	2.8	Q3
6	International Journal of Molecular Sciences	11	409	37.18	9	4.9	Q2
7	Frontiers in Molecular Neuroscience	10	231	23.10	7	3.8	Q2
8	Scientific Reports	8	296	37.00	8	4.6	Q2
9	Brain Research	8	132	16.50	7	2.7	Q3
10	Neuroscience	7	235	33.57	5	3.2	Q3

### 3.4 Author contributions and co-occurrence

Zhuo M is the most prolific author (36 studies, 7.39%), followed by Neugebauer V (16), Chen QY (15), Chen T (13), and Maione S (12) (Table 5). The centrality of these five authors is zero.

**Table 5:** Top 5 authors ranked by publication count and centrality (Based on 487 publications).

Rank	Authors	NP	h_Index	Rank	Authors	Centrality
1	Zhuo M	36	19	1	Zhuo M	0.00
2	Neugebauer V	16	14	2	Neugebauer V	0.00
3	Chen QY	15	9	3	Chen QY	0.00
4	Chen T	13	12	4	Chen T	0.00
5	Maione S	12	12	5	Maione S	0.00

**Figure 5:**
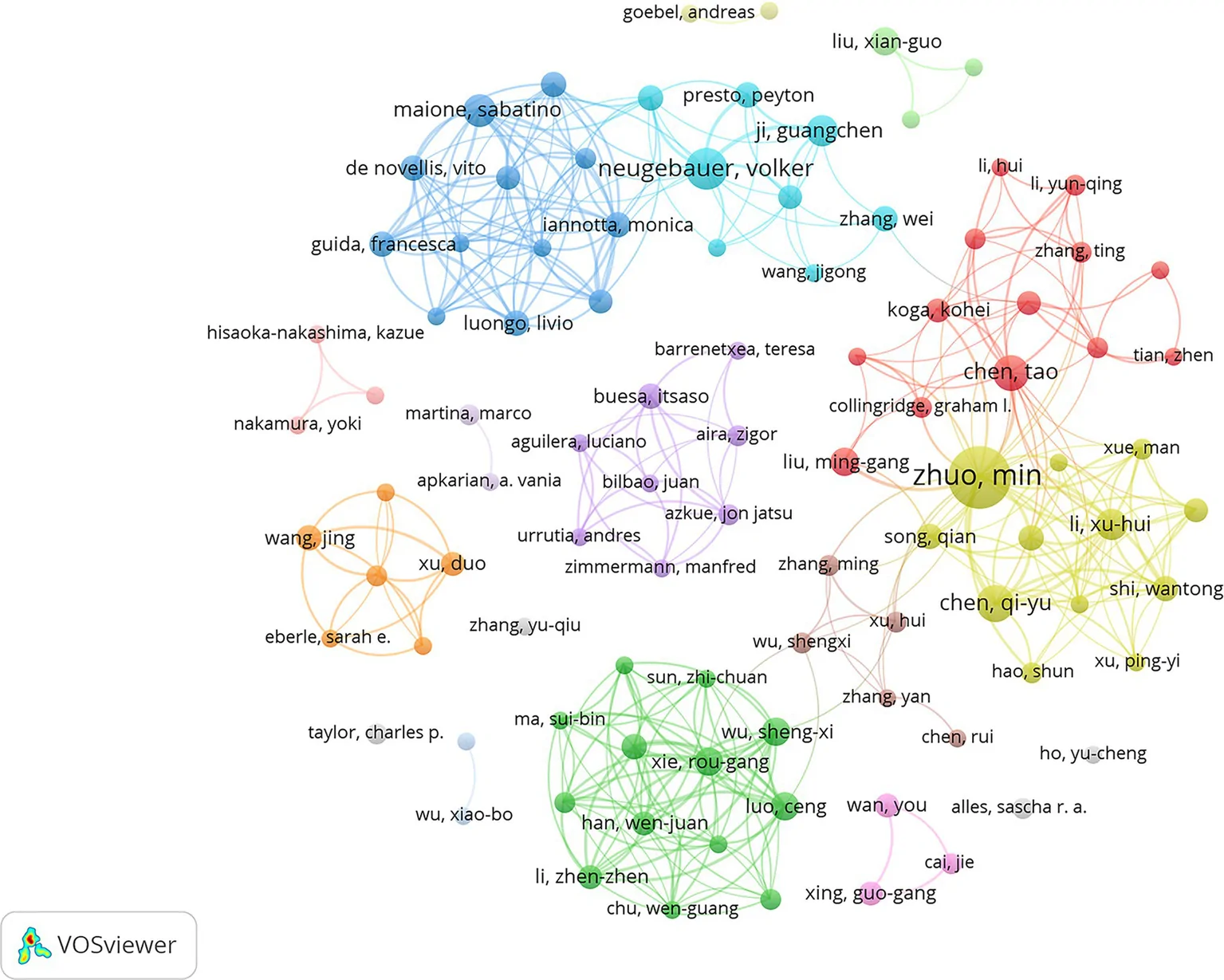
Authors’ collaboration network of research. Created with VOSviewer.

A total of 97 authors published three or more studies, forming 18 collaborative clusters (Figure 5) (19). After excluding single-author clusters, 14 distinct clusters remained. These clusters were categorized into seven research foci based on their themes. (1) ACC and Cortical Mechanisms (Clusters 1, 4): Led by Zhuo M, Collingridge GL, and Chen T. This group investigates synaptic plasticity (LTP/LTD) in the ACC and insular cortex, as well as molecular pathways linking pain and emotion (4, 20–22). (2) Amygdala and Emotional Circuits (Clusters 3, 6): Led by Neugebauer V, Ji G, Maione S, and Boccella S. This group focuses on plasticity of the central amygdala (CeA), endocannabinoid signaling, and stress-related anxiety-like and depression-like behavior in NP (23–27). (3) Spinal and Peripheral Mechanisms (Clusters 2, 7, 9, 10): This category includes subgroups led by Luo C, Xie RG, Wang J, Xu D, Wan Y, Xing GG, and a Japanese team (Hisaoka-Nakashima et al. (28)). Their research covers spinal cord plasticity, ion channels (sodium and calcium), neuron-glial interactions, and novel molecular targets in pain models (6, 28–36). (4) Neuroinflammation and Glial Biology (Clusters 8, 11): Led by Zhang Y, Xu H, Liu XG, and Xin WJ. These groups investigate cytokine signaling (IL-1β, TNF-α), microglial activation, and their effects on pain-related cognitive and emotional comorbidities (8, 37–40). (5) Striatal and Reward Circuitry (Cluster 12): Led by Gao YJ and Wu XB. This group investigates synaptic changes in the nucleus accumbens (NAcc) mediated by NMDA and CCR2 receptor pathways, linking mesolimbic dysfunction to the comorbidity of pain and depression-like behavior (41–43). (6) Cognitive and Translational Neuroscience (Clusters 5, 13, 14): Led by researchers including Apkarian AV, Martina M, Goebel A, and Aira Z. This group utilizes brain imaging, electrophysiology, and human studies to examine brain network alterations, cognitive impairments, and clinical translation in chronic pain (44–48). (7) Independent Influential Scholars: Some researchers have collaborated less extensively with the other authors, yet their individual research continues to exert significant influence. Key examples include Taylor CP (pharmacology of gabapentinoids), Zhang YQ (pain models), Alles SRA (P2X4R-targeted therapies), and Ho YC (pain control) (49–54).

Overall, collaboration among these authors reveals a “multipolar” research landscape. This landscape represents in-depth exploration of diverse topics and holds immense potential for collaboration.

### 3.5 Citation and reference analyses

For details on the 10 most cited studies, see Appendix 1. The most highly cited study was published by Zamponi et al. (18) in 2015, titled “The Physiology, Pathology, and Pharmacology of Voltage-Gated Calcium Channels and Their Future Therapeutic Potential.” This study is cited 910 times and holds the highest average annual citation rate (75.8), making it one of the most influential publications in the field.

**Figure 6:**
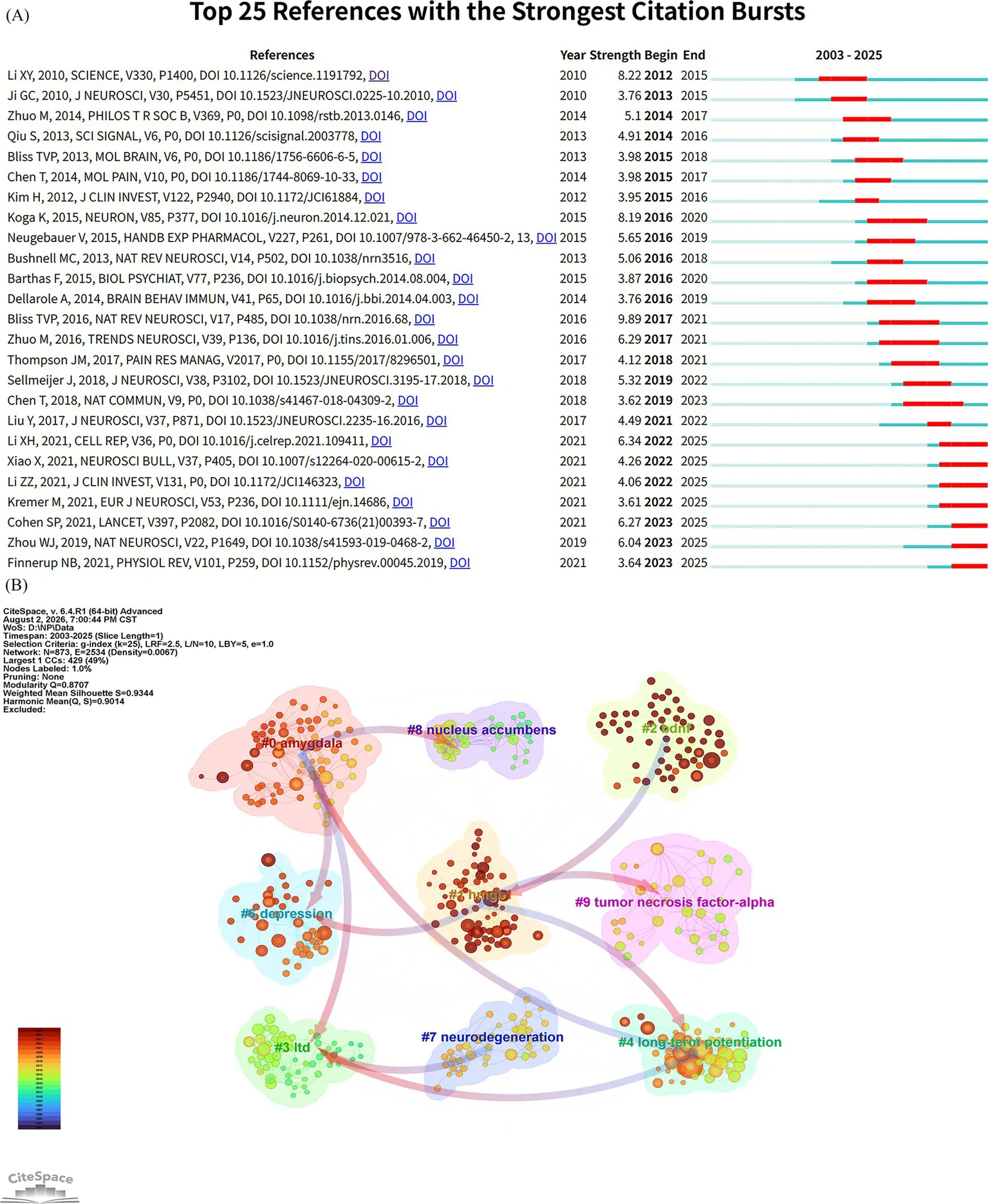
Analysis of references related to synaptic plasticity mechanisms of neuropathic pain with emotional comorbidity. **(A)** The top 25 references with a significant increase in citation frequency. **(B)** Clustering of references according to similarity. Linkage represents connections between different clusters, and the blue groups on the line evolve from the red ones.

A bibliometric analysis revealed a shift in this field from “single-mechanism pain models” to “whole-brain network mechanisms underlying pain comorbidity.” Citation burst analysis (Figure 6A) and cluster network mapping (*Q* = 0.8707, *S* = 0.9344) collectively outline the evolutionary trajectory of this field. First, basic research established the molecular and cellular framework for the emotional dimension of pain. Pioneering studies revealed that sustained activation of PKMζ in the ACC and amygdala-driven dysfunction of the prefrontal cortex are key mechanisms underlying NP and its cognitive-emotional comorbidities (55, 56). Concurrently, shared biochemical pathways between pain and depression—such as brain IDO1 upregulation—were identified, and LTP in the ACC was established as a core cellular model of chronic pain (57, 58). Research scope then expanded from molecular targets to whole-brain neural circuits. Several high-impact reviews have systematically elucidated the mechanisms by which cognition and emotion modulate pain, as well as the mechanisms of synaptic plasticity in the ACC (4, 59). Key empirical advances included: identifying the ACC as a critical hub for pain-induced anxiety- and depression-like behaviors, discovering two distinct LTP forms in ACC mediating sensory versus affective responses, and demonstrating that microglia-dependent TNF-α signaling underlies pain-related depression-like behavior and hippocampal plasticity alterations (20, 60–62). Subsequently, research focus shifted to disease-specific models and translational targets. Studies confirmed the ACC’s top-down modulatory role in spinal transmission and demonstrated that inhibiting excessive ACC activity alleviates negative emotional consequences (63, 64). Refined circuit mechanisms were elucidated, including the dorsal raphe-amygdala-lateral habenula pathway mediating pain-comorbid depression-like behavior, accompanied by dedicated methodological advances for assessing pain-emotion comorbidity (65, 66). The field is now entering a phase of convergence across different research disciplines, with studies becoming more closely integrated with clinical practice. Important review studies in The Lancet and Physiological Reviews summarized what was known about mechanisms and treatments (67, 68). At the same time, as research has progressed, it has been discovered that extracellular matrix proteins (such as laminin β1) play a role in structural changes in synapses, and that oxytocin can inhibit presynaptic LTP in the ACC, thereby helping to alleviate pain and anxiety-like behavior (21, 30). Because of this, the ACC gained renewed attention as a target for neuromodulation (69).

Based on the evolution of the clusters, a temporal analysis of the major clusters (Figure 6B) corroborates this evolutionary trajectory: Early studies of neural circuit maps (Clusters #0 and #8) focused on the amygdala and the NAcc. These studies established a foundational framework for the emotional representation of pain and dysregulation of the reward system (33, 41, 55). In-depth studies of mechanisms centered on the ACC have evolved through distinct stages. Early research emphasized LTD (Cluster #3). This was followed by viewing LTP as the core cellular basis for the comorbidity of pain and emotion (Cluster #4). Recent studies have refined these mechanisms at the molecular level (Cluster #2) (3, 4, 6–8, 29, 70, 71). Neuroimmunological studies (Clusters #7, #9, #6) highlight pro-inflammatory cytokines (TNF-α, IL-1β) as a common pathophysiological bridge linking pain, emotional comorbidity, and cognitive decline (37, 51, 72, 73).

### 3.6 Analysis of co-cited references

**Figure 7:**
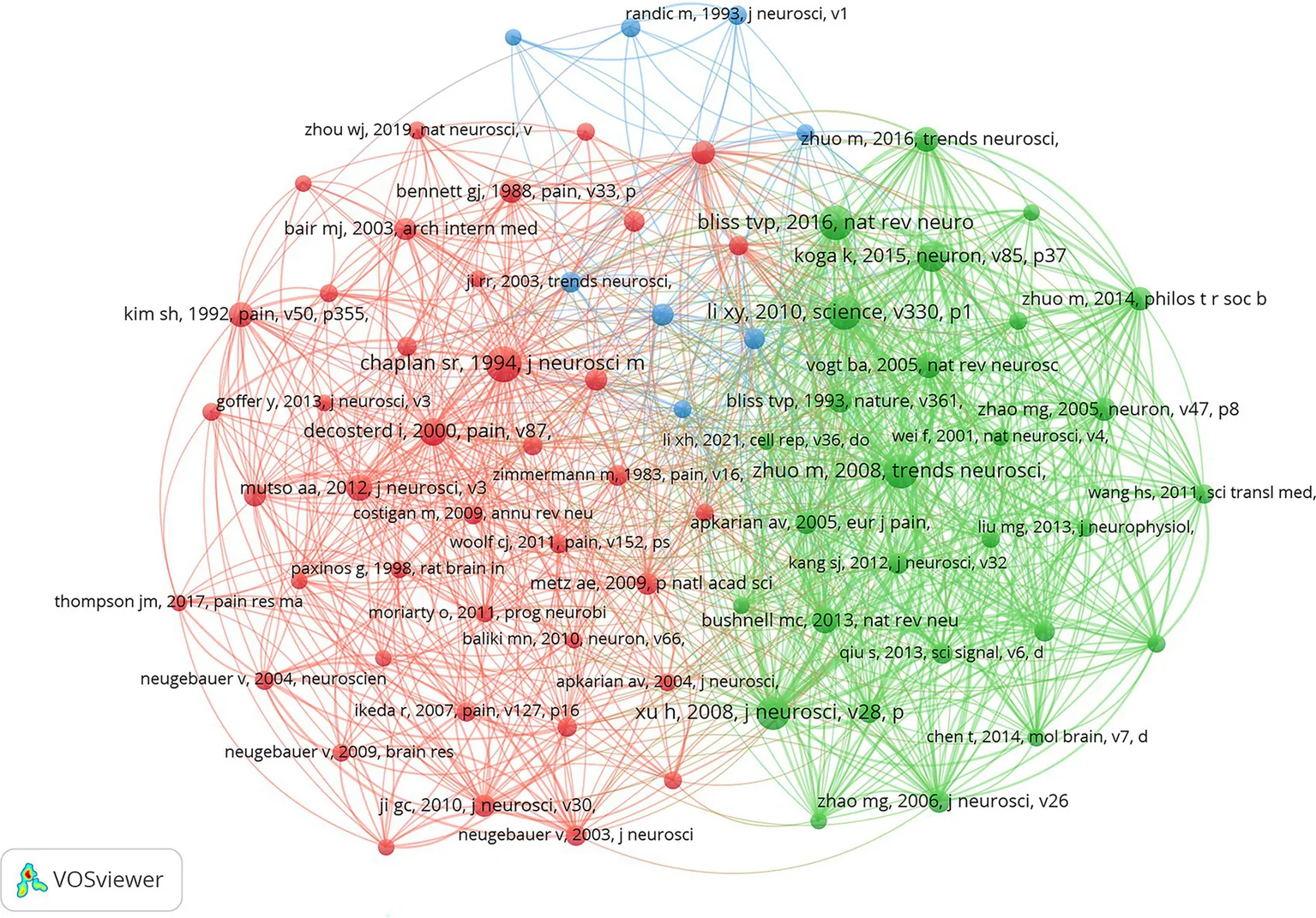
Visualization of a clustering map of co-cited references. Created with VOSviewer.

Co-citation analysis can reveal the knowledge structure of a research field. We used VOSviewer to analyze the 27,363 cited references in the included literature. Appendix 2 lists the top ten most frequently cited studies in this field, covering modeling methods, synaptic mechanisms related to the ACC, pain research from a whole-brain network perspective, and extensions to the cognitive dimension (4, 20, 44, 56, 59, 74–78). After setting a minimum citation threshold of 15, 76 studies were identified and grouped into three clusters (Figure 7).

Cluster 1 (Red): Animal models and translational research. This cluster covers basic models and detection methods used in clinical pain research, including the chronic constriction injury (CCI) model, the spared nerve injury (SNI) model, and the von Frey test for measuring mechanical allodynia (74, 75, 79). Cluster 2 (Green): The relationship between the ACC and synaptic plasticity. This cluster centers on the ACC. The ACC has been established as a central node for chronic pain processing and emotional memory; subsequent studies found that LTP in the ACC sustains chronic pain, while the brain-derived neurotrophic factor (BDNF)-TrkB signaling pathway drives hyperalgesia (3, 56, 58, 76, 77). Cluster 3 (Blue): The dorsal horn of the spinal cord and central sensitization. This cluster elucidates the spinal mechanisms underlying the transition from acute to chronic pain, links LTP to spinal pain mechanisms, incorporates glial cells into the regulatory processes of pain processing, and demonstrates that dysfunction of the noradrenergic system is a factor in the maintenance of pain (80–83).

### 3.7 Keywords co-occurrence analysis

**Figure 8:**
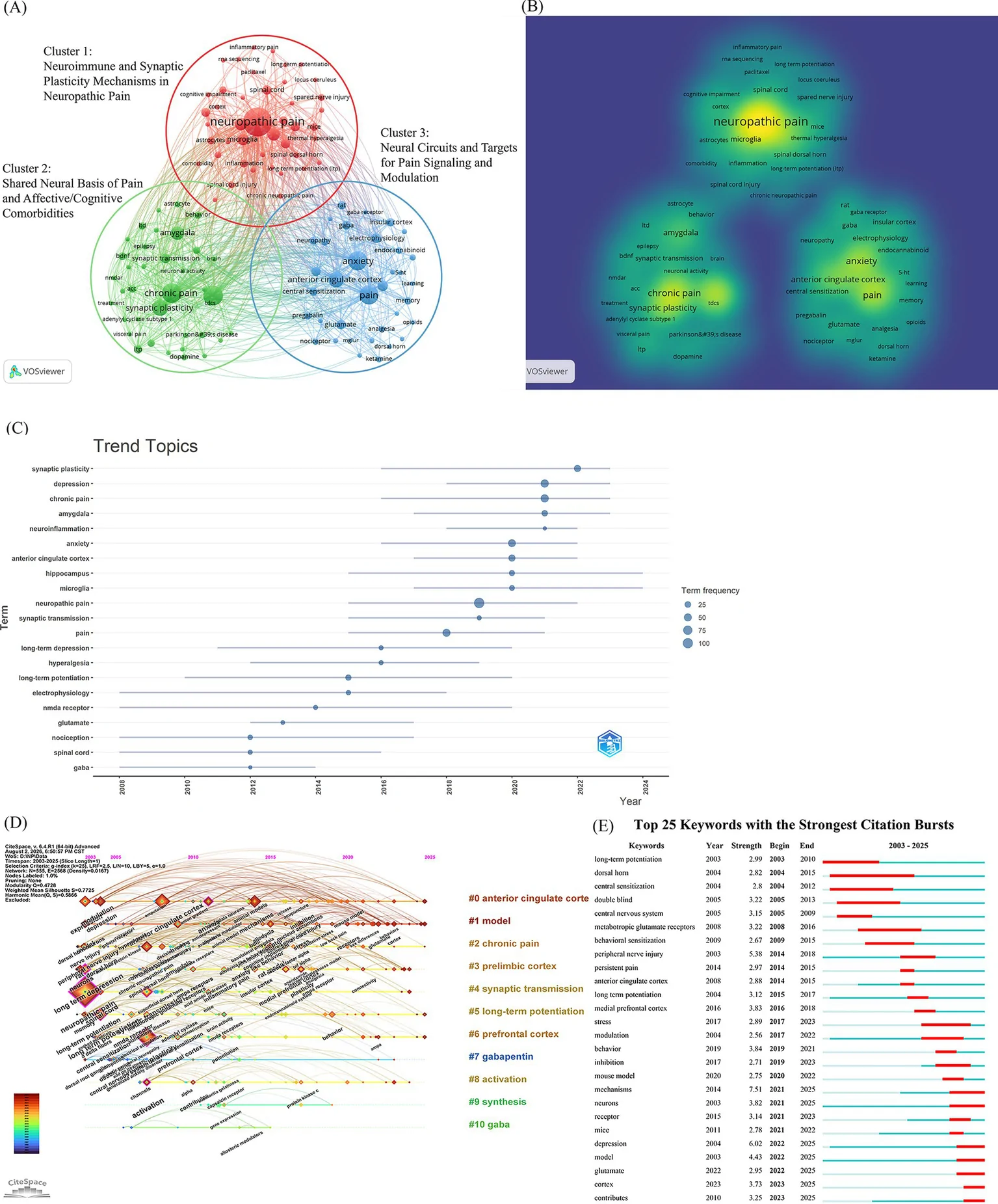
Keyword co-occurrence analysis in synaptic plasticity mechanisms of neuropathic pain with emotional comorbidity research **(A)** Network visualization for keyword co-occurrence analysis (*n* ≥ 3). Keyword co-occurrence network is divided into 3 clusters of different colors according to the evolution of the research hotspots. **(B)** The color of a keyword indicates the average publication time of studies containing that keyword. **(C)** Trend Topics from 2003 to 2025. The timeline illustrates the temporal progression of pivotal research themes within the field. The relative prominence of these research themes undergoes substantial fluctuations over the course of time, with larger nodes denoting elevated frequency and significance. **(D)** Timeline of keyword co-occurrence analysis. The timeline visualizes the temporal evolution of key research topics in the field. **(E)** The 25 keywords with the strongest citation bursts were displayed. The blue line indicates the time axis, with the red segments denoting the start year, end year, and duration of each burst.

Co-occurrence analysis of keywords reveals the thematic structure and development trends in this field. Table 6 lists the top 20 keywords ranked by frequency and centrality. “Neuropathic pain” ranks first in frequency, followed by “synaptic plasticity” and “long term potentiation.” “Long term depression” had the highest centrality, followed by “long term potentiation,” “activation,” and “neuropathic pain.” A total of 115 keywords appeared at least three times and were grouped into three thematic clusters (Figures 8A,B) (19).

**Table 6:** The top 20 keywords in the research ranked by count and centrality (Based on 441 publications).

Rank	Count	Centrality	Year	Keywords	Count	Centrality	Year	Keywords
1	279	0.19	2003	Neuropathic pain	58	0.22	2003	Long term depression
2	124	0.12	2007	Synaptic plasticity	279	0.19	2003	Neuropathic pain
3	88	0.19	2004	Long term potentiation	88	0.19	2004	Long term potentiation
4	78	0.08	2008	Anterior cingulate cortex	42	0.14	2007	Activation
5	59	0.05	2007	Chronic pain	124	0.12	2007	Synaptic plasticity
6	58	0.22	2003	Long term depression	34	0.12	2007	Synaptic transmission
7	52	0.05	2004	Depression	31	0.11	2011	Anxiety
8	42	0.14	2007	Activation	35	0.09	2003	Expression
9	35	0.09	2003	Expression	19	0.09	2004	Central sensitization
10	35	0.05	2014	Mechanisms	78	0.08	2008	Anterior cingulate cortex
11	34	0.12	2007	Synaptic transmission	25	0.08	2015	Rat model
12	33	0.05	2009	Brain	23	0.08	2004	Spinal cord
13	31	0.11	2011	Anxiety	21	0.08	2004	Modulation
14	29	0.05	2003	Model	14	0.06	2004	Dorsal horn
15	25	0.08	2015	Rat model	59	0.05	2007	Chronic pain
16	25	0.05	2003	Neurons	52	0.05	2004	Depression
17	23	0.08	2004	Spinal cord	35	0.05	2014	Mechanisms
18	21	0.08	2004	Modulation	33	0.05	2009	Brain
19	20	0.03	2014	Peripheral nerve injury	29	0.05	2003	Model
20	19	0.09	2004	Central sensitization	25	0.05	2003	Neurons

Cluster 1 (Red), Neuroimmune and synaptic mechanisms in chronic NP, utilizing SNI/CCI models and transcriptomic approaches (75, 79, 84, 85). Cluster 2 (Green), Pain-associated emotional comorbidity and cortico-limbic plasticity, centered on the amygdala, ACC, NAcc, and insula (6–8, 33, 41, 60). Cluster 3 (Blue), Sensory transmission, pharmacology, and behavioral assessment, focused on glutamatergic/GABAergic systems and drugs such as ketamine (an NMDAR antagonist), pregabalin (a calcium channel modulator), and gabapentin (35, 86–89).

Although these three research groups have different focuses within the field, they were closely interrelated. Cluster 1 focuses on disease models and neuroimmune mechanisms; Cluster 2 focuses on emotional comorbidity and the plasticity of limbic system circuits; and Cluster 3 provides foundational methods for behavioral assessment, pharmacological interventions, and electrophysiological studies. There is a close interconnection between Research Cluster 1 and 2, as neuroimmune drivers in the spinal cord and hippocampus accelerate changes in emotion-related circuits. Overall, this keyword network reveals the core pathologies, comorbidity foci, and methodological foundations of the field. The connections among them are also reflected in the trend themes (Figure 8C).

To identify frontier research areas in the study of NP with emotional comorbidity and synaptic plasticity since 2003, keyword clustering analysis was performed using CiteSpace software. Appendix 3 and Figure 8D present the 11 clusters that were ultimately generated. A silhouette value greater than 0.5 generally indicated that the clustering results are reasonable (90). The cluster labels are as follows: #0 anterior cingulate cortex, #1 model, #2 chronic pain, #3 prelimbic cortex, #4 synaptic transmission, #5 long-term potentiation, #6 prefrontal cortex, #7 gabapentin, #8 activation, #9 synthesis, and #10 GABA.

**Figure 9:**
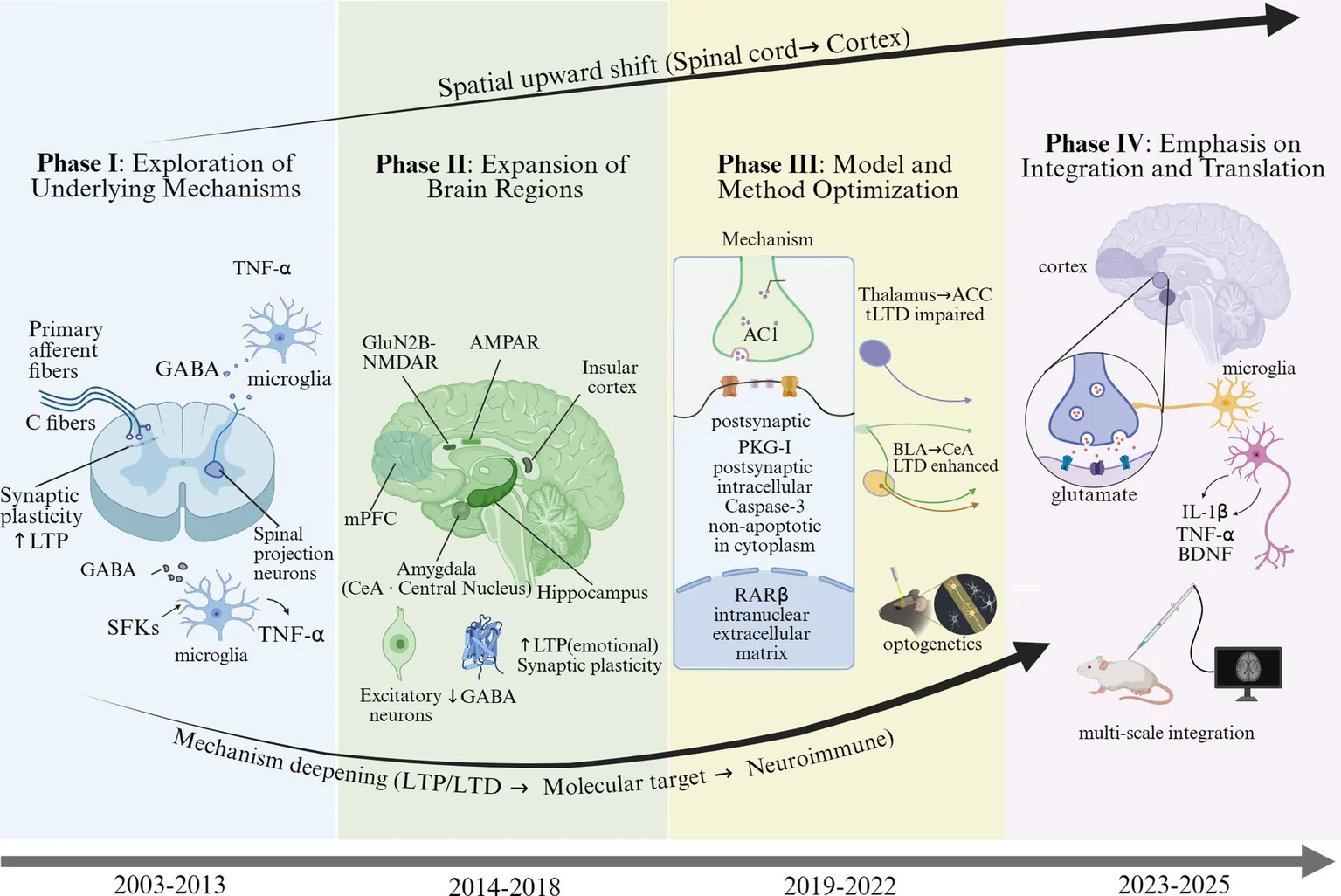
Four key phases in the research on neuropathic pain, emotional comorbidity, and synaptic plasticity, as identified by keyword burst analysis. Created by BioRender. (LTP, Long-term potentiation; GABA, Gamma-aminobutyric acid; TNF-α, Tumor necrosis factor-α; SFKs, Src family kinases; mPFC, Medial prefrontal cortex; CeA, Central nucleus of the amygdala; GluN2B-NMDAR, Glutamate ionotropic receptor NMDA type subunit 2B - N-methyl-D-aspartate receptor; AMPAR, α-Amino-3-hydroxy-5-methyl-4-isoxazolepropionic acid receptor; PKG-I, cGMP-dependent protein kinase I; RARβ, Retinoic acid receptor beta; t-LTD, Timing-dependent long-term depression; AC1, Adenylyl cyclase 1; ACC, Anterior cingulate cortex; IL-1β, Interleukin-1β; BDNF, Brain-derived neurotrophic factor).

Keyword burst analysis identified 25 terms with the strongest citation bursts (Figure 8E) delineating four evolutionary phases (Figure 9).

Exploration of Underlying Mechanisms (2003–2013). This period was defined by several burst keywords: “long-term potentiation” (2003), “dorsal horn” (2004), “central sensitization” (2004), “double-blind” (2005), “central nervous system” (2005), and “metabotropic glutamate receptor” (2008). This phase focused on the basic mechanisms of synaptic plasticity (LTP) and central sensitization at the spinal cord level. Particular attention was paid to the glutamatergic system. Research was dedicated to elucidating the causes of persistent pain at the synaptic and cellular levels (81, 91). Expansion of Brain Regions (2014–2018). Keywords that emerged during this period included “peripheral nerve injury” (2014), “anterior cingulate cortex” (2014), “medial prefrontal cortex” (2016), “stress” (2017), and “modulation” (2017). These keywords indicate that the research focus shifted from the spinal cord to cortical regions such as the ACC and medial prefrontal cortex (mPFC). Related studies revealed the critical role of these cortico-limbic system nodes in emotional comorbidity and cognitive dysfunction associated with chronic pain. The keyword “stress” highlights the bidirectional regulatory mechanisms between the stress system and chronic pain-emotional comorbidity (4, 59). Model and Method Optimization (2019–2022). Burst keywords emerging during this phase included “behavior” (2019), “inhibition” (2019), “mouse model” (2020), “mice” (2021), “mechanisms” (2021, intensity 7.51, the strongest burst in the entire study period), “neurons” (2021), “receptor” (2021), “depression” (2022), “model” (2022), and “glutamate” (2022). This phase is characterized by the proliferation of refined animal models of comorbidity that simultaneously capture NP and depression-like behaviors, enabling dissection of shared neural circuits and molecular targets. The co-emergence of “depression” (intensity 6.02) as the most prominent emotional-comorbidity keyword confirms the central role of pain–depression comorbidity in driving research priorities (72, 92). Emphasis on Integration and Translation (2023–2025). Burst keywords include “cortex” (2023), and “contributes” (2023), while multiple high-intensity bursts from “Model and Method Optimization” continue through 2025. The sustained burst of “cortex” reflects continued emphasis on higher-order brain regions as both explanatory frameworks and therapeutic targets, while the enduring prominence of “depression” affirms that emotional comorbidity remains the dominant translational focus (7, 42, 93–95).

The evolution of high-impact keywords reflects research progress in this field. Spatially, the research focus has shifted from the dorsal horn of the spinal cord to cortical regions such as the ACC and mPFC. Mechanistically, the emphasis has shifted from central sensitization to synaptic plasticity, then to neurotransmitter systems, and most recently to neuroimmune mechanisms involving microglia and neuroinflammation (11, 51, 72). In terms of scope, the field has expanded from nociception to emotional comorbidity (72, 96). Methodologically, the field has evolved toward an integrated approach combining refined animal models with multilevel mechanistic studies (75, 97).

## 4 Discussion

### 4.1 Overall distribution

This study utilized bibliometric methods to identify research hotspots and new frontiers in the field of NP involving emotional comorbidity and synaptic plasticity.

From 2003 to 2025, the total number of studies in this field has grown steadily. China leads in publication output (180 studies), while the United States dominates in citation impact (9,723 citations) and network centrality (0.6). This output-versus-influence gap suggests that Chinese research groups need enhance their international influence (98).

At the institutional level, the top centrality-ranked institutions are the Universite de Bordeaux, CNRS, University of California system, David Geffen School of Medicine at UCLA and Air Force Medical University. Core authors (Zhuo M, Neugebauer V, Chen QY, Chen T, Maione S) exhibit zero centrality. This may reflect several non-mutually-exclusive factors: the multipolar cluster structure of the co-authorship network, CiteSpace’s Pathfinder pruning algorithm, author-name disambiguation limitations in WoSCC records, and genuine technical specialization across laboratories. Centrality values alone should not be interpreted as direct evidence of limited collaboration. In this field, core journals include the Journal of Neuroscience, PAIN, Molecular Brain, Neuropharmacology, and Molecular Pain which warrant close attention from researchers. Looking ahead, fostering connections among collaborative networks across countries, institutions, and authors is crucial for advancing the field.

### 4.2 Evolution of research priorities

An analysis of keyword trends reveals four stages in the evolution of this research topic. Phase I (2003–2013) focused on the dorsal horn of the spinal cord, where nerve injury-induced C-fiber LTP and the microglial Src-family kinase–TNF-α axis were identified as core mechanisms of central sensitization, while emotional comorbidity received little attention (9, 35, 38, 99). Phase II (2014–2018) marked a spatial shift toward the cerebral cortex and limbic system, as synaptic LTP in the ACC was shown to link pain with emotion and motivation, while attenuated GABAergic inhibition in the CeA was found to drive anxiety-like behavior; Subsequent work extended these findings to the insular cortex, prefrontal cortex, NAcc, and hippocampus, targeting NMDAR subunits (GluN2B) and AMPA receptors (4, 6, 8, 33, 44, 100). Phases III–IV (2019–2025) brought an increasing number of mechanistic insights: molecular targets including AC1, PKG-I, Caspase-3, and RARβ were implicated in the LTP/LTD balance and the regulation of affective behaviors; pathway-specific plasticity was elucidated (thalamus–ACC for sensory symptoms, BLA–CeA for emotional symptoms); optogenetics and conditional knockout approaches have established causal evidence at the circuit level; non-pharmacological strategies, such as electroacupuncture, environmental enrichment, and multi-omics analyses, have become tools for biomarker discovery and therapeutic development (7, 29, 31, 70, 85, 95, 101–104). The evolution of research focus across these four stages reflects the gradual refinement of anatomical focus and the depth of understanding of underlying mechanisms.

### 4.3 Research hotspots and knowledge gaps

Current hotspots center on four domains: region-specific synaptic plasticity in the limbic system, encompassing receptor subtypes, signaling molecules, and epigenetic modifications that drive region-specific LTP/LTD and consequent sensory or emotional dysfunction (6, 8, 10, 29, 105). Research has also shifted from individual brain regions to neural circuits, aiming to elucidate the underlying mechanisms of pain-emotion signal integration. Key questions focus on the connections between sensory (spinal cord, thalamus), emotional (amygdala, ACC), and cognitive (prefrontal cortex) regions, as well as the mechanisms by which chronic stress amplifies negative effects through circuit-level plasticity. Concurrently, research has emphasized how activated microglia and astrocytes modulate neuronal excitability and synaptic plasticity by releasing cytokines, chemokines, and neurotrophic factors. Based on these findings, novel pharmacological targets, neuromodulatory strategies, and non-pharmacological interventions are currently being actively developed (2, 10, 31, 102, 103, 106–109).

Despite these advances, several specific gaps warrant attention. First, the synaptic mechanisms underlying pain-emotion comorbidity in the insular cortex and NAcc remain comparatively understudied relative to the ACC and amygdala, representing an opportunity for targeted investigation. Second, sex differences in synaptic plasticity related to pain–emotion comorbidity are not well represented in the current bibliometric landscape, suggesting that, at the level of the specific keywords analyzed here, there is still insufficient explicit focus on this variable. Third, longitudinal study designs are not captured as a distinct keyword or cluster in the present analysis, consistent with a field in which cross-sectional experimental designs predominate in the indexed literature. Fourth, the translation of preclinical circuit-manipulation findings (e.g., optogenetic rescue of ACC LTD) to human clinical applications remains an unmet challenge requiring biomarker-driven bridging strategies. Fifth, although exercise is low-cost and clinically accessible, direct evidence that it alleviates pain–emotion comorbidity by modulating synaptic plasticity remains sparse. Preliminary findings indicate that voluntary running reverses ACC synaptic degeneration and restores LTP in osteoarthritis models, and that environmental enrichment restores AMPA/NMDAR function in the mPFC and promotes hippocampal BDNF signaling and neurogenesis (103, 109, 110). However, causal evidence linking these synaptic changes to relief of pain-associated affective behaviors is lacking.

## 5 Limitations of research

Several limitations should be considered when interpreting these findings. First, bibliometric metrics reflect research attention and knowledge linkages, not biological causality or clinical efficacy; co-citation clustering identifies intellectual structure through shared citation patterns but does not independently validate the mechanistic claims made in the cited literature. Second, WoSCC provides structured citation metadata suitable for network and burst analyses, whereas PubMed records lack equivalent cited-reference data and citation counts; consequently, bibliometric network analyses were restricted to 441 WoSCC publications, with 46 PubMed-unique records contributing only to descriptive statistics, and publications indexed exclusively in Scopus or other databases were not captured. Third, the analysis was restricted to English-language articles and reviews as an explicit study-design choice, meaning that non-English publications, regional journals, and locally indexed research may be underrepresented. Fourth, citation-based metrics are subject to temporal bias (recent publications have less time to accumulate citations) and structural bias (journal visibility and field size influence citation counts independently of research quality), and should not be interpreted as direct measures of study quality. Fifth, residual author-name disambiguation errors—particularly for common East Asian surnames—and institutional-name inconsistencies may affect author- and institution-level metrics. Finally, network metrics and cluster assignments are sensitive to analytical parameter choices (thresholds, pruning algorithms, clustering resolution) and should be understood as one valid representation among several possible configurations of the same underlying data.

## 6 Conclusion

This bibliometric analysis maps the knowledge landscape of NP, emotional comorbidity, and synaptic plasticity research across 487 publications (2003–2025). The sustained growth in output and interdisciplinary collaboration reflects accelerating interest in plasticity-based mechanisms of pain-emotion comorbidity, spanning neuroscience, psychiatry, immunology, molecular biology, computational science. Research has transitioned from spinal cord-centric models to cortico-limbic circuit integration, with the ACC, amygdala, and NAcc emerging as central hubs. Current trends advance along two complementary axes: deepening mechanistic resolution (molecular → cellular → circuit) while broadening scope to encompass systemic interactions. These findings provide a structured overview for future explanatory and interventional studies aimed at elucidating shared synaptic mechanisms between pain and emotional comorbidity.

## Data Availability

The original contributions presented in the study are included in the article/Supplementary material, further inquiries can be directed to the corresponding authors.

## Footnotes

1
https://bibliometric.com/

## Glossary

AC1: adenylyl cyclase 1
ACC: anterior cingulate cortex
α₂δ: voltage-gated calcium channel α₂δ subunit
AMPA: α-amino-3-hydroxy-5-methyl-4-isoxazolepropionic acid
AMPAR: α-amino-3-hydroxy-5-methyl-4-isoxazolepropionic acid receptor
BDNF: brain-derived neurotrophic factor
BLA: basolateral amygdala
CBT: cognitive-behavioral therapy
CCI: chronic constriction injury
CCL2: C-C motif chemokine ligand 2
CCR2: C-C chemokine receptor type 2
CeA: central nucleus of the amygdala
CNS: central nervous system
DBS: deep brain stimulation
DRG: dorsal root ganglion
EEG: electroencephalography
ERK: extracellular signal-regulated kinase
FTO fat mass and obesity-associated protein
GABA: gamma-aminobutyric acid
GluA1: AMPA receptor subunit 1
GluN2B: glutamate ionotropic receptor NMDA type subunit 2B
HMGB1: high mobility group box 1
HPA: hypothalamic–pituitary–adrenal axis
IDO1: indoleamine 2,3-dioxygenase
IL-1β: interleukin-1β
KCC2: K^+^-Cl^−^ Cotransporter 2
LA: lateral amygdala
LFP: local field potential
LTD: long-term depression
LTP: long-term potentiation
m6A: N^6^-methyladenosine
mGluR5: metabotropic glutamate receptor 5
mPFC: medial prefrontal cortex
NAcc: nucleus accumbens
NMDA: N-methyl-D-aspartate
NMDAR: N-methyl-D-aspartate receptor
NP: neuropathic pain
P2X4R: P2X7R P2X purinoceptor 4/7
PFC: prefrontal cortex
PKA: protein kinase A
PKG-I: cGMP-dependent protein kinase I
PKMζ: protein kinase Mζ
RARβ: retinoic acid receptor *β*
ROS: reactive oxygen species
SNI: spared nerve injury
tDCS: transcranial direct current stimulation
tLTD: timing-dependent long-term depression
TMS: transcranial magnetic stimulation
TNF-α: tumor necrosis factor-α
VGCCs: voltage-gated calcium channels
WoSCC: Web of science core collection
