# Supplementary material for: Synaptic plasticity in affective comorbidities of neuropathic pain: a bibliometric analysis based on WoSCC and PubMed (2003–2025)
Source: Front Neurol. 2026 Sep 3;17:1916513. doi: 10.3389/fneur.2026.1916513 (PMC13581774; doi:10.3389/fneur.2026.1916513)
Supplement: Supplementary file 1 [file Table_1.DOCX]

**Appendix 1**  The top 10 most cited studies

| **Rank** | **Title** | **First author** | **Journal** | **Year** | **TC** | **Descriptions** |
| --- | --- | --- | --- | --- | --- | --- |
| 1 | The Physiology, Pathology, and Pharmacology of Voltage-Gated Calcium Channels and Their Future Therapeutic Potential | Zamponi GW | Pharmacological Reviews | 2015 | 910 | This review summarizes the subtypes, physiological functions, and pharmacological significance of voltage-gated calcium channels, and explores the potential for developing novel analgesic and neuroprotective drugs targeting specific subtypes. |
| 2 | Plasticity in the human central nervous system | Cooke SF | Brain | 2006 | 846 | This review summarizes evidence for synaptic plasticity (such as LTP) in the human central nervous system and discusses how non-invasive stimulation techniques can be used to modulate plasticity for the treatment of depression and Parkinson’s disease. |
| 3 | Synaptic plasticity in the anterior cingulate cortex in acute and chronic pain | Bliss TVP | Nature Reviews Neuroscience | 2016 | 676 | It focuses on how synaptic plasticity in the ACC mediates chronic pain and its associated emotional components |
| 4 | The Link between Depression and Chronic Pain: Neural Mechanisms in the Brain | Sheng JY | Neural Plasticity | 2017 | 587 | The study analyzed the neurobiological basis of the comorbidity between chronic pain and depression, emphasizing that the neuroplastic changes underlying both conditions share significant overlapping mechanisms. |
| 5 | X-ray structure of dopamine transporter elucidates antidepressant mechanism | Penmatsa A | Nature | 2013 | 529 | By analyzing high-resolution crystal structures of dopamine transporters bound to antidepressants, the molecular mechanism by which these drugs inhibit neurotransmitter reuptake has been elucidated. |
| 6 | Pharmacology and mechanism of action of pregabalin: the calcium channel alpha2-delta (alpha2-delta) subunit as a target for antiepileptic drug discovery | Taylor CP | Epilepsy Research | 2007 | 487 | It provides a detailed explanation of how pregabalin exerts its anticonvulsant and analgesic effects by binding to the α₂δ subunit of calcium channels, thereby reducing neurotransmitter release. |
| 7 | Down-regulation of the potassium-chloride cotransporter KCC2 contributes to spasticity after spinal cord injury | Boulenguez P | Nature Medicine | 2010 | 478 | The study revealed that downregulation of the KCC2 cotransporter following spinal cord injury leads to the failure of inhibitory neurotransmission, thereby triggering pathological spasticity. |
| 8 | Abnormalities in hippocampal functioning with persistent pain | Mutso AA | Journal of Neuroscience | 2012 | 422 | It has been demonstrated that chronic pain leads to a reduction in hippocampal volume and functional abnormalities, which explains why patients often experience learning difficulties and emotional deficits. |
| 9 | Morphological and functional reorganization of rat medial prefrontal cortex in neuropathic pain | Metz AE | Proceedings of the National Academy of Sciences | 2009 | 365 | It has been found that neuropathic pain leads to morphological reorganization and enhanced synaptic function in neurons of the mPFC, which may be associated with the cognitive impairment caused by chronic pain. |
| 10 | Ca2+ channel alpha2delta ligands: novel modulators of neurotransmission | Dooley DJ | Trends in Pharmacological Sciences | 2007 | 317 | This review summarizes how α₂δ ligands, as a novel class of neurotransmitter modulators, treat pain and anxiety disorders by inhibiting excessive neuronal excitation. |

**Appendix 2** The top 10 highly co-cited references

| **Rank** | **Title** | **Citation** | **Year** |
| --- | --- | --- | --- |
| 1 | Quantitative assessment of tactile allodynia in the rat paw | 63 | 1994 |
| 2 | Cortical excitation and chronic pain | 61 | 2008 |
| 3 | Synaptic plasticity in the anterior cingulate cortex in acute and chronic pain | 60 | 2016 |
| 4 | Alleviating neuropathic pain hypersensitivity by inhibiting PKMzeta in the anterior cingulate cortex | 60 | 2010 |
| 5 | Presynaptic and postsynaptic amplifications of neuropathic pain in the anterior cingulate cortex | 58 | 2008 |
| 6 | Coexistence of two forms of LTP in ACC provides a synaptic mechanism for the interactions between anxiety and chronic pain | 45 | 2015 |
| 7 | Spared nerve injury: an animal model of persistent peripheral neuropathic pain | 40 | 2000 |
| 8 | Pain and emotion interactions in subregions of the cingulate gyrus | 37 | 2005 |
| 9 | Cognitive and emotional control of pain and its disruption in chronic pain | 36 | 2013 |
| 10 | Abnormalities in hippocampal functioning with persistent pain | 34 | 2012 |

**Appendix 3** Keyword cluster analysis

| **Cluster ID** | **Size** | **Silhouette** | **Mean Year** | **Label** | **Other keywords** |
| --- | --- | --- | --- | --- | --- |
| 0 | 73 | 0.774 | 2015 | anterior cingulate cortex | acupuncture; post-long-term potentiation; p2x(7) receptor |
| 1 | 70 | 0.635 | 2018 | model | circuits; antagonists; electroacupuncture; neurogenesis |
| 2 | 67 | 0.741 | 2012 | chronic pain | neuronal network; peripheral nerve injury; basolateral amygdala; cognition |
| 3 | 65 | 0.754 | 2014 | prelimbic cortex | chronic neuropathic pain; anandamide; protein-protein interactions; fluorescence polarization |
| 4 | 59 | 0.743 | 2012 | synaptic transmission | nociception; spinal cord; electrophysiology; reactive oxygen species |
| 5 | 53 | 0.818 | 2012 | long-term potentiation | long term potentiation; alzheimers disease; anxiety-like behavior; randomized controlled trial |
| 6 | 43 | 0.811 | 2012 | prefrontal cortex | low frequency stimulation; eeg; central sensitization; synaptic plasticity |
| 7 | 33 | 0.915 | 2007 | gabapentin | calcium channel; pregabalin; anticonvulsant; presynaptic |
| 8 | 32 | 0.756 | 2015 | activation | capsaicin receptor; spasticity; central nervous system; primary afferents |
| 9 | 16 | 0.922 | 2013 | synthesis | opioid system; passion flower; insomnia; ups |
| 10 | 14 | 0.945 | 2009 | gaba | gaba(c) receptor ligands; gaba aminotransferaseinhibitors; gaba(a) receptor ligands; gaba uptake inhibitors |
